# Quantitative and semi-quantitative computed tomography analysis of interstitial lung disease associated with systemic sclerosis: A longitudinal evaluation of pulmonary parenchyma and vessels

**DOI:** 10.1371/journal.pone.0213444

**Published:** 2019-03-12

**Authors:** Mariaelena Occhipinti, Silvia Bosello, Leuconoe Grazia Sisti, Giuseppe Cicchetti, Chiara de Waure, Tommaso Pirronti, Gianfranco Ferraccioli, Elisa Gremese, Anna Rita Larici

**Affiliations:** 1 Department of Clinical and Experimental Medicine, University of Florence, Florence, Italy; 2 Rheumathology Division, Fondazione Policlinico Universitario “A. Gemelli” IRCCS, Rome, Italy; 3 Department of Public Health–Section of Hygiene, Catholic University of Sacred Heart, Rome, Italy; 4 Institute of Radiology, Pole of Imaging, Laboratory and Infectivology Sciences, Diagnostic Imaging Area, Fondazione Policlinico Universitario “A. Gemelli” IRCCS, Rome, Italy; 5 Department of Experimental Medicine, University of Perugia, Perugia, Italy; Keio University, JAPAN

## Abstract

**Objectives:**

To evaluate interstitial lung disease associated with systemic sclerosis (SSc-ILD) and its changes during treatment by using quantitative analysis (QA) compared to semi-quantitative analysis (semiQA) of chest computed tomography (CT) scans. To assess the prognostic value of QA in predicting functional changes.

**Materials and methods:**

We retrospectively selected 35 consecutive patients with SSc-ILD with complete pulmonary functional evaluation, Doppler-echocardiography, immunological tests, and chest CT scan at both baseline and follow-up after immunosuppressive therapy. CT images were analyzed by two chest radiologists for semiQA and by a computational platform for texture analysis of ILD patterns (CALIPER) for QA. Concordance between semiQA and QA was tested. Traction bronchiectasis severity was scored. Analysis of ROC curves was performed.

**Results:**

Seventy CT scans were analyzed and QA failed in 4/70 scans. Thus, the final population included 31/35 patients (51.3±12.1 years). QA had a weak-to-good concordance with semiQA (ICC reticular:0.275; ICC ground-glass:0.667) and QA correlated better than semiQA (r = -0.3 to -0.74 vs r = -0.3 to -0.4) with functional parameters. Both methods correlated with traction bronchiectases score and pulmonary artery diameter at CT. A pulmonary artery diameter ≥29mm distinguished patients with lower lung volumes and ILD extent greater than 39% (*p*<0.001). Changes in QA patterns during treatment were not accurate (AUC: 0.50 to 0.70; *p*>0.05) in predicting disease progression as assessed by functional parameters, whereas variation in total lung volume at QA accurately predicted changes in the composite functional respiratory endpoint with FVC% and DLco% (AUC = 0.74; 95%CI: 0.54 to 0.93; p = 0.03).

**Conclusions:**

Pulmonary QA of CT images can objectively quantify specific patterns of ILD changes during treatment in patients with SSc-ILD. Changes in QA patterns do not correlate with functional changes, but variation in total lung volume at QA accurately predicted changes in the composite functional respiratory endpoint with FVC% and DLco%. Pulmonary artery diameter at CT reflects the interstitial involvement, identifying patients with more severe prognosis.

## Introduction

Systemic sclerosis (SSc) is a connective tissue disease with a variety of clinical presentations, ranging from involvement restricted to the skin and peripheral angiopathy to rapidly progressive forms affecting internal organs. Lungs are one of the most commonly affected organs, with both parenchyma and pulmonary vessels involvement [1].

Diffusing capacity of the lung for carbon monoxide (DLco) and forced vital capacity (FVC) levels have traditionally been used as measures of lung disease severity and reductions in both parameters have been associated with increased mortality in the interstitial lung disease associated with SSc (SSc-ILD) [2, 3]. Recently Goh et al. proposed a composite functional end-point with FVC and DLco for predicting survival in patients with SSc-ILD [3] and a decade ago the same group proposed a radiological score together with FVC to distinguish “limited” versus “extensive” disease [4, 5]. This approach is restricted to evaluate patient prognosis and is limited to a semi-quantitative analysis (semiQA) of chest computed tomography (CT) images, susceptible to observer bias, inter-reader, and intra-reader variability [6]. These limits represent a critical obstacle especially when evaluating the response to therapy in daily practice, which is the real unmet need in the assessment of SSc population [7, 8].

Quantitative analysis (QA) of chest CT images has recently become feasible with the advent of computational platforms able to perform a fully automated lung texture analysis that can identify and quantify lung fibrosis [9, 10]. One of the available platforms, called Imbio Lung Texture Analysis (LTA) (Imbio LLC, Minneapolis, MN), is CE (Conformité Européenne) mark certified and approved for clinical settings. Imbio LTA identifies and quantifies the lung CT patterns of ILD (i.e. ground-glass, reticular, honeycombing) based on an anatomo-pathological validation [10]. The objectivity, reproducibility, and the full automatism of this technique make QA a desirable tool to evaluate lung damage and response to therapy in SSc patients in clinical trials and eventually in daily clinical practice [11].

Therefore, we aimed to evaluate extent and patterns of SSc-ILD and their changes over time by using QA on chest CT scans, to assess the concordance of QA and semiQA scores, and to ascertain whether QA correlates more strongly than semiQA with functional data and vascular CT parameters. Finally, we aimed to assess the prognostic value of QA in predicting pulmonary function changes and to compare the clinical response with the radiological quantitative response.

## Materials and methods

### Patients selection

This is a retrospective study on human subjects, approved by the local ethics committee (Comitato Etico Fondazione Policlinico universitario “Agostino Gemelli”- Università Cattolica del Sacro Cuore). Informed consent was waived for this retrospective evaluation. Thirty-five patients with SSc-ILD admitted to the Scleroderma Clinic of the study center were selected. Inclusion criteria were those proposed by the European League Against Rheumatism (EULAR) and the American College of Rheumatology for the diagnosis of SSc [12] and patients were classified as “limited” and “diffuse” type according to LeRoy classification [13]. Exclusion criteria were the presence of chronic obstructive pulmonary disease, history of lung cancer, age less than 18 years old, and long-term oxygen therapy (due to poor performance of these patients at pulmonary function tests–PFT, often left incomplete, and difficulties in assessing DLco). The baseline evaluation occurred between October 2012 and October 2014 and patients were followed-up to December 2016.

Disease duration was defined as years since the onset of Raynaud’s phenomenon or the first non-Raynaud’s phenomenon manifestation. Patients with disease duration since the first non-Raynaud’s phenomenon manifestation less than 3 years were considered to have an early disease.

The included patients underwent PFT, Doppler-echocardiography, immunological tests, and unenhanced chest CT scan before and after therapy. Immunosuppressive therapies were either rituximab or conventional immunosuppressive drugs (azathioprine, mycophenolate, or cyclophosphamide).

### Pulmonary function tests

Spirometric evaluation with flow-sensing computer-connected spirometers (Biomedin Spirometer) provided data on pulmonary function, in particular measurements of predicted values of forced vital capacity (FVC%), total lung capacity (TLC%), and residual volume (RV%) were acquired adopting the American Thoracic Society/European Respiratory Society (ATS/ERS) standards [14]. To ensure reproducibility of each parameter the measurement of lung volumes was repeated at least two times for each parameter and the best value was recorded. FVC% less than or equal to 79% with normal forced expiratory volume in 1s was defined as restrictive lung disease. Measurements of carbon monoxide diffusing capacity (DLco) were also performed to measure the integrity of the blood-gas barrier. DLco measurement was considered valid if the inspiratory volume of the single breath was at least 90% of the vital capacity.

### Immunological tests

ANA, anti-ENA with anti-Scl-70 antibodies (antitopoisomerase antibodies), ACA (anticentromere antibodies), C3 and C4 were measured. ANA antibodies were determined by indirect immunofluorescence using Hep-2 cells as substrates and autoantibodies specificities were further assessed by ELISA (Shield, Dundee, UK).

### Echocardiography

Two-dimensional echocardiography Doppler examination was performed and left ventricular ejection fraction was measured on apical four- and two-chamber views using the Simpson method. Pulmonary artery systolic pressure (PASP) was estimated using the tricuspid regurgitation velocity and the Bernoulli equation. Mean PASP on echocardiography in our cohort was 28.6±6.4 mmHg and 8 patients (25.8%) presented a PASP ≥35 mmHg. Considering the importance of increased PASP on echocardiography in patients with scleroderma, we decided to evaluate all patients with DETECT algorithm. According to STEP 2 of DETECT algorithm (total risk points >35) five patients underwent right heart catheterization. In none of these five patients a diagnosis of pulmonary arterial hypertension was confirmed on right heart catheterization, despite a PASP ≥35 mmHg on echocardiography.

### CT scan technique and image analysis

Chest CT scan was performed with a Philips Brilliance-64 scanner (Philips Medical Systems) periodically calibrated. Acquisition parameters were set to 120 kVp, 212 mAs, 0.5s rotation time, pitch 1.0. The raw data were processed to create two sets of 1mm thick axial slices, one using standard reconstruction algorithm (Convolution Kernel D-Philips Medical) and one using a high-resolution reconstruction algorithm (Convolution Kernel B-Philips Medical).

*QA* (quantitative analysis) was performed in the set of images with standard reconstruction algorithm by using Imbio LTA (based on the algorithm of CALIPER—Computer Aided Lung Informatics for Pathology Evaluation and Rating), a computational platform for the near-real-time characterization and quantification of lung parenchymal patterns on CT scans [10, 15]. Lung parenchymal patterns include Normal, Hyperlucent, Ground-glass (GG), Reticular, and Honeycombing. Imbio LTA provided the total lung volume, relative volumes, and absolute volumes of lung patterns, and their regional distribution within three different lung zones (upper, middle, lower) in each lung. These data were provided in the output images, in a quantitative table, and in a glyph with color-coded sectors whose size is proportional to the percentage distribution of that pattern in each lung zone. The relative volumes of GG and Reticular patterns were added to represent the total burden of disease. The platform provided a fast output of DICOM series as well as a pdf file in which each lung pattern was colored differently, allowing the evaluation of disease extent, composition, and location at a glance. Moreover, the final report permitted an easy comparison between baseline and follow-up. Each QA was performed automatically in up to 20’.

*SemiQA* (semiquantitative analysis) was performed on the images reconstructed with high-resolution kernel by two chest radiologists (MO, ARL), at first independently from each other and then after 3 months in consensus. To score the interstitial parenchymal disease we used the international Goh score at 5 lung levels: 1) origin of great vessels; 2) main carina; 3) pulmonary venous confluence; 4) halfway between the third and fifth section; 5) immediately above the right hemi-diaphragm [4, 5]. The CT variables scored were total disease extent, extent of GG pattern, extent of reticular pattern, and coarseness of reticulation. Images were analyzed at lung window setting. Each semiQA was performed by the radiologists independently in 3 to 5’ whereas in consensus reading in 5 to 10’. In consensus reading the two radiologists assigned a severity grade to traction bronchiectasis, according to the score reported in literature used for assessment of idiopathic pulmonary fibrosis [16]. The score took into account the average degree of airway dilatation within areas of fibrosis as well as the extent of dilatation within the lobe, with a final score of: 0 = none, 1 = mild, 2 = moderate, 3 = severe.

The widest short-axis diameter of the main *pulmonary artery* (PA) on axial sections at the level of PA bifurcation and the widest short-axis diameter of the ascending aorta (Ao) at the same level of PA measurement were computed with electronic calipers and their ratio (PA/Ao) was calculated. Mediastinal window setting was used to measure vascular CT parameters. A cut-off of 29 mm was used to define pulmonary hypertension on CT, according to the literature data that report a specificity of 89% and a sensitivity of 87% for this cutoff [17, 18].

### Changes in functional parameters and CT scores during follow-up

Clinically disease progression was defined as FVC and DLco composite, consisting of either a relative FVC decline from baseline >10% or an FVC decline of 5–9% in association with a DLco decline of >15% [3, 19, 20]. An improvement was defined either an FVC increase from baseline >10% or an FVC increase of 5–9% in association with a DLco improvement of >15%. All other outcomes were defined as stability.

Radiologically at QA four different cutoffs were explored (2%, 5%, 10%, 20% [5, 21]) to define a change from baseline. Progression of disease was defined if the reduction in %Normal pattern was greater than the cutoff value, improvement if an increase in %Normal pattern greater than the cutoff value was observed, and stability if none of the above conditions applied.

### Data analysis

Mean and standard deviation (SD) were used to describe quantitative variables. Absolute and relative frequencies were used to describe qualitative variables.

Intraclass correlation coefficient (ICC) was calculated to compare the concordance between semiQA scores of independent readers and between semiQA scores obtained by consensus reading and QA scores [22].

Pearson r correlation coefficient was used to compare QA scores and semiQA scores with PFT, DLco%, PASP, traction bronchiectases score, and vascular CT parameters, as well as to compare the variations of FVC% and DLco% between baseline and follow-up (ΔFVC and ΔDLco).

T-test was used to assess differences in LTA patterns between patients with a PA diameter ≥29mm or <29mm [23].

Paired t-test was used to compare means of lung parameters assessed by QA at both baseline and follow-up.

Cohen k coefficient was used to assess the concordance between functional and radiological treatment responses using the above-mentioned cutoffs.

Analysis of ROC curves and the corresponding areas under the curve (AUC) were performed to assess the predictability of FVC% and DLco% decline (as detailed above) from variations in QA patterns (Δ%Normal, Δ%GG, Δ%Reticular, Δ%GG+%Reticular). An AUC value greater than 0.70 was deemed indicative for accuracy.

A *p* value <0.05 was deemed statistically significant and data analysis was performed by using IBM SPSS Statistics 22.

## Results

Patients included 27 women (77.1%) and 8 men (22.9%), aged 51.8±11 years. A total of 70 CT scans were analyzed, 35 at each time point. QA failed in 4/70 scans (6%) due to technical reasons, “field of view too small” in one case and “airways larger than expected size” in three cases. The final population included 31 patients (aged 51.3±12.1 years; women: 52.6±11.9, men: 45.5±12.1) with complete clinical and imaging evaluations at both baseline and follow-up. Because of progression of skin and/or lung involvement 12/31 (38.7%) patients received treatment with rituximab and 19/31 (61.3%) patients received conventional immunosuppressive drugs. Most patients had diffuse disease (23/31, 74.2%) and were positive for anti-Scl-70 antibodies (26/31, 83.9%). Table 1 reports clinical, functional and imaging data.

**Table 1:** Clinical and radiological characteristics at baseline of the 31 patients with SS-ILD.

Age, mean ±SD, yy		51.3±12.1
Disease duration, mean ±SD, yy		8.9±4.8
M/ F, n (%)		6/25 (19.4/80.6)
Early disease/Long disease, n (%)		4/27 (12.9/87.1)
Diffuse/limited skin involvement, n (%)		23/8 (74.2/25.8)
Follow-up, mean ±SD, months		26.0±12.6
Anti-topoisomerase I/ACA/ANA* antibodies, n (%)		26/0/5 (83.9/0.0/16.1)
FVC%, mean ±SD		82.5±22.2
TLC%, mean ±SD		77.0±19.6
RV%, mean ±SD		73.5±21.2
DLco%, mean ±SD		48.4±19.9
Kco, mean ±SD		59.0±21.1
FVC ≤79%, n (%)		13 (41.9)
FVC ≤60%, n (%)		5 (16.1)
PASP, mean ±SD, mmHg		28.6±6.4
PASP ≥35 mmHg, n (%)		8 (25.8)
Vascular parameters	PA diameter	26.2 (±2.6)
	Ao diameter	30.1 (±3.7)
	PA/Ao ratio	0.9 (±0.1)
SemiQA (consensus reading)	%Disease Extent	31.4 (±18.9)
	%GG	22.9 (±13.3)
	%Reticular	8.4 (±7.8)
	Coarseness reticulation	4.4 (±2.4)
	Traction bronchiectasis score	9.54 (±1.1)
QA (Imbio LTA)	%Normal	76.4 (±18.4)
	%Hyperlucent	0.4 (±1.8)
	%GG	18.3 (±16.6)
	%Reticular	4.7 (±4.5)
	%Honeycombing	0
	%GG+Reticular	23.0 (±18.5)
	Lung volume (L)	3.8 (±0.8)

Data are reported as mean ± standard deviation. Percentages express the relative volume of each pattern to the whole lung.

ACA = anti-centromere antibodies, ANA = anti-nuclear antibodies, Ao = ascending aorta, DLco = diffusing lung capacity for carbon monoxide, F = female, FVC = forced vital capacity, GG = ground-glass, Kco = carbon monoxide transfer coefficient, L = liters, PA = pulmonary artery, LTA = lung texture analysis, M = male, PASP = pulmonary artery systolic pressure, QA = quantitative analysis, RV = residual volume, SD = standard deviation, semiQA = semiquantitative analysis, TLC = total lung volume.

*ANA positivity without specificities.

### SSc-ILD lung patterns and their regional distribution

GG was the dominant pattern at both QA and semiQA (18.3% and 22.9%, respectively—Table 1), whereas honeycombing was absent in this population. At semiQA both %GG and %Reticular extent grew from apex to base, along with coarseness of reticulation (Table 2). Similarly, QA showed a predilection for lower lung zones for both GG and Reticular patterns, with an apex-to-base crescendo of alterations (Table 3).

**Table 2:** Regional distribution of lung patterns at semiQA as performed by consensus reading in each lung level at both baseline and follow-up.

		Global score	*p*	Level 1	*p*	Level 2	*p*	Level 3	*p*	Level 4	*p*	Level 5	*p*
**%Disease extent**	Baseline	31.4 ±18.9	.64	12.1 ±11.9	.19	16.8 ±14.5	.16	32.7 ±22.9	.63	41.9 ±28.1	.72	53.4 ±28.1	.70
	Follow-up	32.5 ±21.1		16.5 ±19.0		21.6 ±21.3		31.5 ±22.6		40.8 ±27.7		52.1 ±27.9	
**%GG**	Baseline	23.0 ±13.3	.92	11.8 ±11.4	.23	16.8 ±14.5	.20	32.6 ±22.8	.59	41.6 ±28.0	.69	52.6 ±28.5	.55
	Follow-up	23.2 ±15.0		15.5 ±17.7		21.0 ±20.2		31.1 ±22.6		40.3 ±27.4		50.6 ±26.8	
**%Reticular**	Baseline	8.4 ±7.8	**.*04***	2.7 ±5.3	.08	4.8 ±6.9	.11	13.5 ±15.6	.21	18.4 ±20.2	.70	23.9 ±22.5	.12
	Follow-up	9.3 ±8.1		4.0 ±6.0		5.8 ±7.9		14.7 ±15.3		18.9 ±18.9		26.8 ± 24.3	
**Coarseness reticulation**	Baseline	3.1 ±2.5	.15	0.4 ±0.7	**.*01***	0.5 ±0.5	.10	0.7 ±0.5	.66	0.8 ±0.7	.74	1.0 ±0.7	.74
	Follow-up	3.5 ±2.4		0.6 ±0.6		0.7 ±0.6		0.8 ±0.5		0.8 ±0.6		1.0 ±0.6	

Values for each lung level (levels are described in the main text) are expressed as means ± standard deviation. p values refers to differences between baseline and follow-up calculated by using paired t-test.

GG = ground-glass.

**Table 3:** Regional distribution of lung patterns at QA in each lung zone at both baseline and follow-up.

		Total lung	*p*	Left lung	*p*	LU	*p*	LM	*p*	LL	*p*	Right lung	*p*	RU	*p*	RM	*p*	RL	*p*
**%Normal**	Baseline	76.4 ±18.4	0.754	75.9 ±19.0	0.602	90.6 ±13.1	0.391	79 ±21.5	0.793	51.1 ±31.7	0.586	76.6 ±18.9	0.869	88.4 ±14.5	0.608	78.1 ±21.3	0.832	55.9 ±31.9	0.953
	Follow-up	75.7 ±21.1		74.3 ±21.5		88.3 ±19.5		78.1 ±23.4		48.5 ±31.9		76.3 ±22.2		87.1 ±19.4		77.7 ±24.3		55.7 ±32.8	
**%HyperL**	Baseline	0.4 ±1.8	0.734	0.4 ±1.8	0.877	0.5 ±2.2	0.738	0.4 ±2.2	1	0.4 ±1.4	0.394	0.5 ±2.0	0.726	0.5 ±1.7	0.928	0.4 ±2.0	0.724	0.7 ±2.3	0.398
	Follow-up	0.3 ±0.9		0.4 ± 1.3		0.7 ± 2.2		0.4 ±1.5		0.1 ±0.4		0.4 ± 0.8		0.4 ±1		0.3 ± 0.8		0.3 ± 0.8	
**%GG**	Baseline	18.3 ±16.6	0.433	18.7 ±17.7	0.339	6.0 ±10.0	0.288	16.9 ±20.2	0.640	39.5 ±31.2	0.318	18.3 ±17.2	0.542	7.6 ±11.3	0.417	17.6 ±20.0	0.572	36.5 ±30.5	0.894
	Follow-up	19.8 ±18.4		21.2± 19.5		8.1 ±15.7		18.4 ±21.8		44.0 ±30.5		19.4 ±18.9		9.1 ± 14.7		18.6 ±21.8		37.0 ±29.9	
**%Reticular**	Baseline	4.7 ±4.5	0.404	4.9 ±4.1	0.334	2.8 ±4.1	0.965	3.6 ±3.9	0.651	8.9 ±7.6	0.194	4.4 ±5.0	0.687	3.4± 4.9	0.880	3.6± 4.5	0.531	6.7 ±7.7	0.817
	Follow-up	3.9 ±3.4		4.1 ±3.0		2.7 ±4.2		3.2 ± 2.5		7 ±4.4		4.1 ± 4.1		3.3 ±5.4		3.1 ±3.5		6.3 ±6.8	
**%HC**	Baseline	0	0.325	0	0.325	0	-	0	-	0	0.083	0	0.184	0	-	0	0.325	0.1 ±0.3	0.057
	Follow-up	0.0 ±0.2		0.0 ± 0.2		0		0		0.1 ±0.3		0.1 ±0.4		0		0.0 ±0.2		0.4 ±1.1	
**% GG+** **Reticular**	Baseline	23.0 ±18.6	0.752	26.6 ±19.4	0.587	8.8 ±13.4	0.417	20.5 ±22.0	0.758	48.4 ± 31.8	0.579	22.8 ±19.2	0.758	11.1± 14.7	0.588	21.3 ±21.6	0.832	43.2 ±32.0	0.984
	Follow-up	23.7 ±21.4		25.2 ±21.9		10.9 ±19.6		21.6 ±23.6		51.0 ±32.1		23.5 ±22.3		12.4 ±19.9		21.7 ±24.4		43.3 ±32.4	

Values for each lung zone (upper, middle, lower) and are expressed as means ± standard deviation. p values refers to differences between baseline and follow-up calculated by using paired t-test. Percentages express the relative volume of each pattern to the whole lung.

GG = ground-glass; HC = honeycombing; HyperL = hyperlucent; LL = left lower zone; LM = left middle zone; LU = left upper zone; RL = right lower zone; RM = right middle zone; RU = right upper zone.

### Concordance of QA and semiQA scores

By analyzing all CT scans, QA scores compared to semiQA scores obtained by consensus reading had weak concordance for Reticular pattern (ICC:0.275, 95%CI: 0.029 to 0.489, *p* = 0.015), moderate concordance for GG pattern (ICC:0.667, 95%CI: 0.502 to 0.785, *p*<0.001) and good concordance for total disease extent (ICC:0.766, 95%CI: 0.640 to 0.852, *p*<0.001).

SemiQA scores between independent readers had fair concordance for GG pattern (ICC:0.549, 95%CI: 0.291 to 0.721, *p*<0.001) and good concordance for total disease extent (ICC:0.761, 95%CI: 0.572 to 0.863, p<0.001) as well as for Reticular pattern (ICC:0.881, 95%CI: 0.810 to 0.927, p<0.001).

### Imaging scores and pulmonary function

A negative correlation was found between PFT and semiQA scores (r = -0.3 to -0.4, *p*<0.001) (Table 4). The correlations were stronger between PFT and QA scores (r = -0.3 to -0.74, *p*<0.001) (Table 4). The strongest correlations were between %GG or %GG+Reticular and TLC% (r = -0.74 and -0.72, respectively) and between %GG or %GG+Reticular and FVC% (both r = -0.71).

**Table 4:** Pearson’s r correlation coefficients between semi-quantitative (SemiQA) and quantitative (QA) scores with pulmonary function, arterial measurements, and traction bronchiectasis score.

		SemiQA scores				QA scores				
		%Disease Extent	%GG	%Reticular	Coarseness reticulation	%Normal	%GG	%Reticular	%GG+%Reticular	Lung Volume
FVC%	r	-0.455	-0.369	-0.484	-0.454	**0.698**	**-0.719**	-0.380	**-0.709**	0.637
	p	*<* .*001*	.*003*	*<* .*001*	*<* .*001*	*<* .*001*	*<* .*001*	.*003*	*<* .*001*	*<* .*001*
TLC%	r	-0.522	-0.447	-0.518	-0.470	**0.712**	**-0.736**	-0.427	**-0.721**	**0.748**
	p	*<* .*001*	.*001*	.*001*	*<* .*001*	*<* .*001*	*<* .*001*	.*001*	*<* .*001*	*<* .*001*
RV%	r	-0.481	-0.438	-0.458	-0.331	0.579	-0.573	-0.533	-0.578	0.606
	p	.*001*	.*003*	.*002*	.*026*	*<* .*001*	*<* .*001*	*<* .*001*	*<* .*001*	*<* .*001*
DLco%	r	-0.360	-0.272	-0.419	-0.357	0.414	-0.404	-0.301	-0.416	0.268
	p	.*004*	.*034*	.*001*	.*005*	.*001*	.*001*	.*018*	.*001*	.*037*
Kco	r	-0.395	-0.290	-0.489	-0.297	0.295	-0.301	-0.192	-0.296	-0.145
	p	.*003*	.*035*	*<* .*001*	.*031*	.*032*	.*028*	.169	.*032*	.299
PA diam.	r	0.372	0.312	0.377	0.096	-0.363	0.382	0.263	0.387	-0.256
	p	.*003*	.*014*	.*003*	.457	.*004*	.*002*	.*039*	.*002*	.*045*
Ao diam.	r	0.066	0.056	0.066	-0.056	-0.155	0.125	0.180	0.144	0.015
	p	.620	.673	.621	.674	.242	.346	.172	.275	.908
PA/Ao	r	0.258	0.215	0.264	0.097	-0.148	0.198	0.025	0.178	-0.228
	p	.051	.105	.*045*	.471	.269	.135	.853	.182	.085
PASP	r	0.301	0.329	0.139	-0.346	-0.229	0.192	0.325	0.242	-0.056
	p	.079	.053	.428	.*042*	.186	.268	.057	.162	.750
TB score	r	0.57	0.44	0.64	0.30	-0.39	0.39	0.25	0.40	-0.06
	p	*<* .*001*	*<* .*001*	*<* .*001*	.*031*	.*001*	.*001*	.*04*	.*001*	.65

Statistically significant correlation coefficients (p<0.05) are in *italics*, whereas the strongest correlations are in **bold**.

Ao = ascending aorta, DLco = diffusing lung capacity for carbon monoxide, FVC = forced vital capacity, GG = ground-glass, Kco = transfer coefficient, PA = pulmonary artery trunk diameter, PA/Ao = pulmonary artery trunk to ascending aorta ratio, PASP = pulmonary artery systolic pressure, RV = residual volume, TB = traction bronchiectasis, TLC = total lung capacity.

### Traction bronchiectases

Traction bronchiectases were present in 25/31 (81%) patients and 50/62 scans, with a severity score at baseline of 9.54, worsening to 10.8 at follow-up. Traction bronchiectases score significantly correlated with QA and semiQA scores, as detailed in Table 4. In particular, Traction bronchiectases score inversely correlated with %Normal and positively correlated with %GG and %Reticular at both QA and semiQA. Traction bronchiectases score correlated with DLco (r = -0.34, p < .01), TLC(r = -0.30, p = .02), and PA diameter (r = 0.34, p < .01), but not with PASP (r = 0.05, p = .98).

### Vascular analysis

Among vascular parameters (PA diameter, Ao diameter, PA/Ao ratio, PASP) PA diameter was significantly correlated with both QA and semiQA scores (Table 4). No significant correlations were found between QA scores and Ao diameter, PA/Ao ratio, PASP. A PA diameter ≥29mm distinguished patients with higher QA scores in %GG, %Reticular, %GG+Reticular (*p*<0.001) (Table 5), while the value of echocardiographic PASP did not (*p*>0.05). Those patients with a PA diameter greater than or equal to 29mm had %GG+Reticular greater than 39% (*p*<0.001) and lower lung volumes (*p* = 0.003) than patients with a PA diameter less than 29mm.

**Table 5:** Differences in quantitative analysis (QA) patterns of SSc-ILD according to pulmonary artery diameter.

***QA Parameter***	**PA ≥29mm**	**PA <29mm**	***p***
***%Normal***	60.8 ± 19.5	81.4 ± 16.9	**<0.001**
***%GG***	32.6 ± 16.5	14.3 ± 15.2	**<0.001**
***%Hyperlucent***	0.1 ± 0.2	0.5 ± 1.6	0.346
***%GG+Reticular***	38.9 ± 19.5	18.0 ± 17.1	**<0.001**
***%Reticular***	6.2 ± 3.9	3.6 ± 3.8	**0.022**
***%Honeycombing***	0.1 ± 0.2	0 ± 0	0.090
***Lung volume***	3.3 ± 0.6	3.9 ± 0.8	**0.003**

Data are reported as mean ± standard deviation.

GG = ground glass, PA = pulmonary artery, QA = quantitative analysis.

### Follow-up evaluation and outcome prediction

Patients were followed-up for 26 (±13) months. At follow-up semiQA and QA showed a predilection of GG and Reticular patterns for lower lung zones, with an apex-to-base crescendo of alterations (Tables 2 and 3). Between baseline and follow-up no significant differences in pattern extent across the lung zones were observed at QA (Table 3), whereas at semiQA the global score of %Reticular pattern significantly increased (p = .04, Table 2). Differences in total lung volume were not significant either (3.83±0.77L at baseline vs 3.75±0.79L at follow-up, *p* = 0.345). Among the 25 patients who had traction bronchiectases, in 17 patients the traction bronchiectasis score was stable between baseline and follow-up, whereas in 8 patients the score upgraded.

ΔFVC had a weak positive correlation with ΔDLco (r = 0.40, *p* = 0.025).

**Fig 1:**
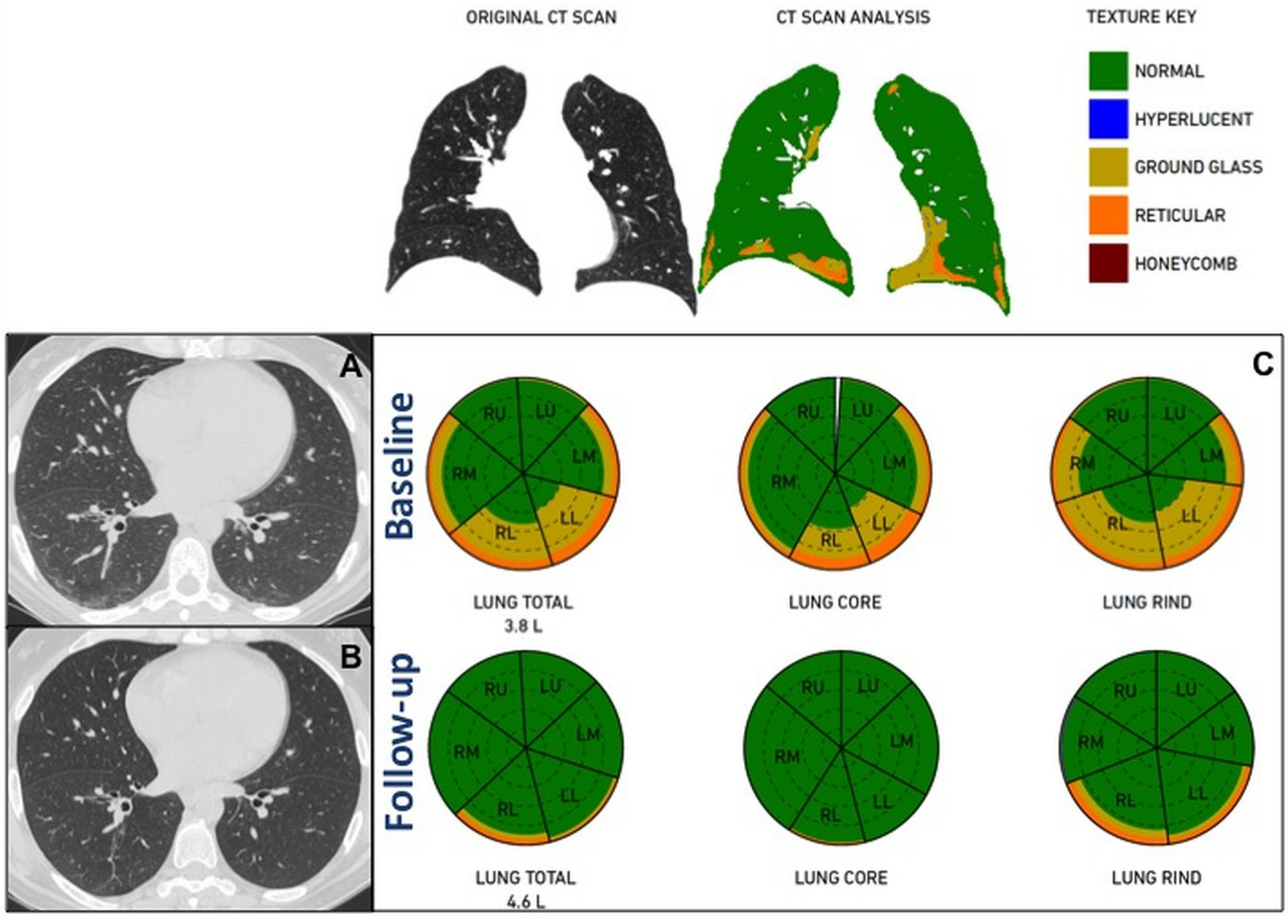
44 years-old woman showing an improvement of ILD at QA after therapy with rituximab. PFT revealed an increase in FVC% (87% to 101%) and a decrease in DLco% (69% to 50%). Axial chest CT scans at the same level at baseline (A) and follow-up (B) show reduction of the diffuse and bilateral ground-glass opacities with a complete disappearance of the superimposed subpleural reticulation in the lower lobes. QA analysis performed by Imbio LTA shows an increase in total lung volumes (3.8L to 4.6L) and in %Normal pattern (74% to 97%) with a reduction in %GG (20% to 1%) and %Reticular (6% to 2%) patterns, as shown in the glyphs (C).

**Fig 2:**
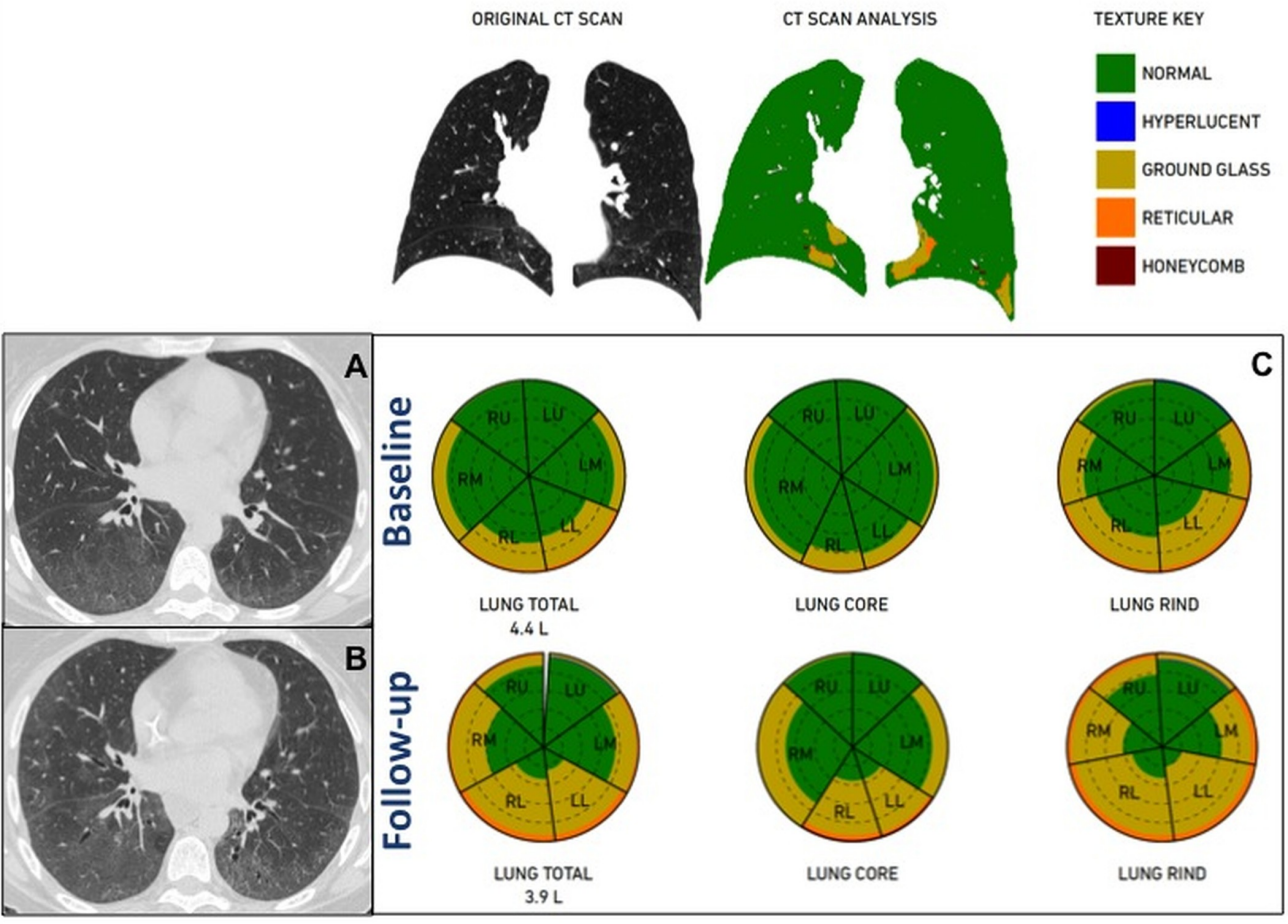
46 years-old woman showing a progression of ILD at QA after therapy with rituximab. PFT revealed a mild increase in FVC% (90% to 97%) and a stability in DLco% (27%). Axial chest CT scans at the same level at baseline (A) and follow-up (B) show increase of the diffuse and bilateral ground-glass opacities more evident in the subpleural regions in both lower lobes associated with fine intralobular reticulation. QA analysis performed by Imbio LTA shows a reduction in total lung volumes (4.4L to 3.9L) and in %Normal pattern (83% to 61%) with an increase in %GG (15% to 35%) and %Reticular (2% to 4%) patterns, as summarized in the glyphs (C).

Table 6 shows the results of clinical and radiological modification during follow-up after therapy related to different cutoffs (2%, 5%, 10%, 20%) of changes in %Normal at QA, with cases of improvement (Fig 1), stability and progression (Fig 2). The definition of significant clinical modification by composite endpoint with FVC and DLco did not correlate with the radiological score by QA for all the different cutoff values tested: at 2% κ = -0.020, *p* = 0.853; at 5% κ = -0.016, p = 0.885; at 10% κ = 0.040, *p* = 0.618; at 20% κ = 0.046, *p* = 0.232. By increasing the cutoff of change in %Normal (i.e. from 2% to 20%) the improvement rate declined with a parallel increase in the progression rate.

**Table 6:** Clinical and radiological modifications in response to therapy according to groups of therapy (either rituximab or other conventional immunosuppressive drugs) and to different cut-offs (2%, 5%, 10%, 20%) of changes in %Normal at QA.

	Therapy	Improvement	Stability	Progression
Composite functional endpoint FVC+DLco	RTX	4/12 (33.3%)	6/12 (50%)	2/12 (16.7%)
	Others	6/19 (31.6%)	4/19 (21%)	9/19 (47.4%)
QA Δ%Normal >2%	RTX	6/12 (50%)	0	6/12 (50%)
	Others	7/19 (36.8%)	0	12/19 (63.2%)
QA Δ%Normal >5%	RTX	5/12 (41.7%)	0	7/12 (58.3%)
	Others	5/19 (26.3%)	1/19 (5.3%)	13/19 (68.4%)
QA Δ%Normal >10%	RTX	3/12 (25%)	2/12 (16.7%)	7/12 (58.3%)
	Others	2/19 (10.5%)	4/19 (21%)	13/19 (68.4%)
QA Δ%Normal >20%	RTX	1/12 (8.3%)	0	11/12 (91.7%)
	Others	0	0	19/19 (100%)

DLco = diffusing capacity for carbon monoxide, FVC = forced vital capacity, QA = quantitative analysis, RTX = rituximab.

**Fig 3:**
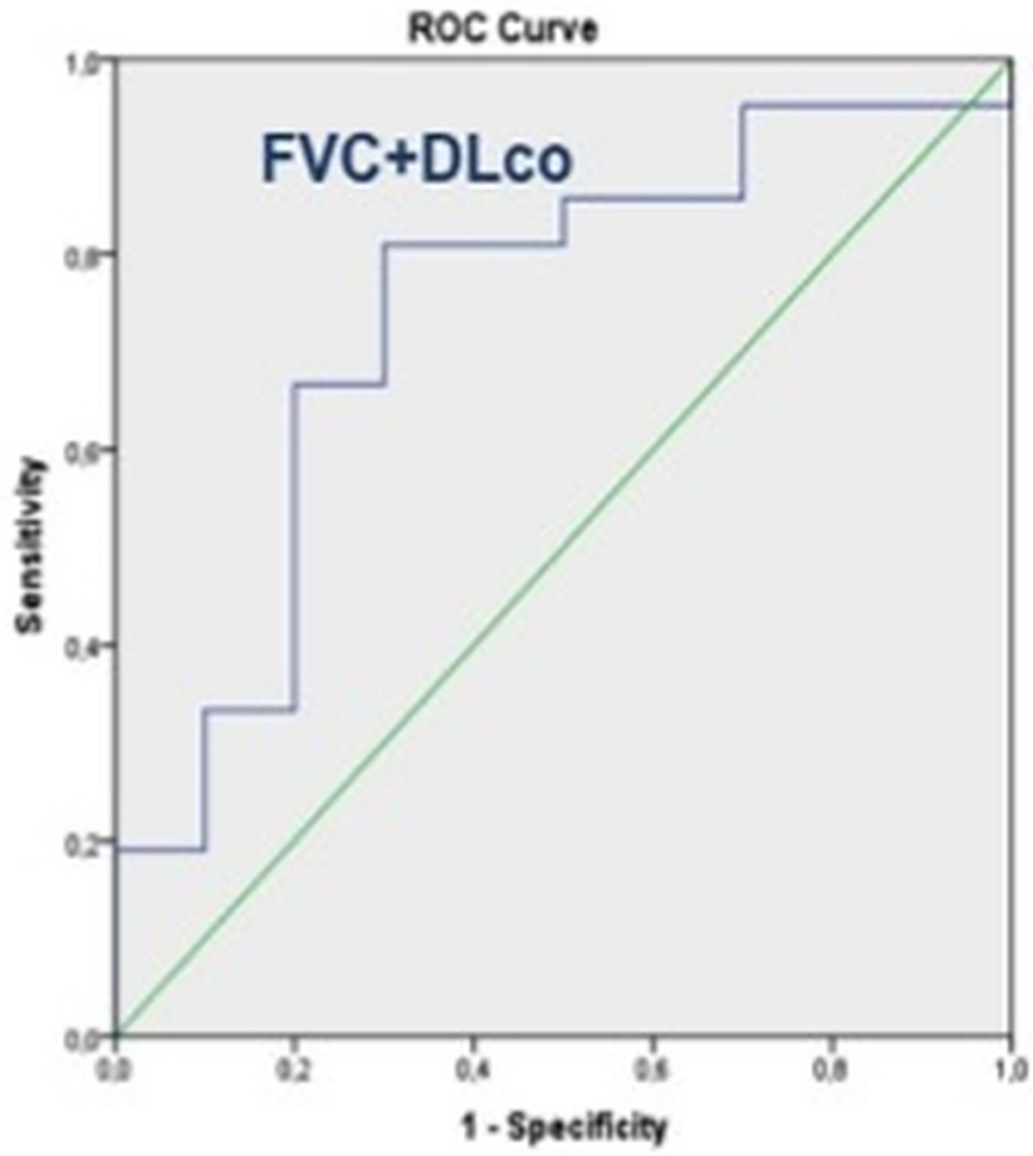
ROC curve of lung volume at QA to predict the FVC and DLco composite end-point. The variation in total lung volume calculated by Imbio LTA accurately predicted the composite functional respiratory endpoint with FVC% and DLco% (AUC = 0.74; 95%CI: 0.54 to 0.93; p = 0.03), but not changes in FVC% and DLco% alone (see also Table 6).

Variations in QA patterns between baseline and follow-up (Δ%Normal, Δ%GG, Δ%Reticular, Δ%GG+Reticular) were not accurate (AUC:0.50 to 0.70; *p*>0.05) in predicting disease progression as assessed by PFT. However, variation in total lung volume calculated by Imbio LTA accurately predicted the composite functional respiratory endpoint with FVC% and DLco% (AUC = 0.74; 95%CI: 0.54 to 0.93; *p* = 0.03) (Table 7) (Fig 3). A reduction in total lung volume greater than 7.18% predicted a progression of disease as of a combined decline in FVC% and DLco% with a sensitivity of 81% and a specificity of 70%. Conversely, Imbio LTA did not accurately predict changes in FVC% and DLco% alone (Table 7).

**Table 7:** ROC curves and associated AUC relative to progression of SSc-ILD.

**Δ**	**Δ FVC + DLco**		**FVC <79% at follow-up**		**Δ FVC%**		**Δ DLco%**	
	AUC	p	AUC	p	AUC	p	AUC	p
**%Normal**	0.51 (0.29–0.74)	.89	0.59 (0.38–0.81)	.372	0.43 (0.18–0.68)	.629	0.47 (0.22–0.71)	.777
**%GG**	0.54 (0.29–0.79)	.726	0.66 (0.44–0.88)	.165	0.57 (0.28–0.86)	.626	0.45 (0.17–0.72)	.677
**%Reticular**	0.70 (0.51–0.89)	.078	0.62 (0.42–0.82)	.262	0.70 (0.53–0.88)	.156	0.63 (0.41–0.85)	.258
**%GG+Reticular**	0.52 (0.30–0.75)	.826	0.61 (0.40–0.81)	.318	0.54 (0.29–0.80)	.760	0.51 (0.25–0.76)	.946
**Lung Volume**	0.74 (0.54–0.93)	**.035**	0.74 (0.57–0.92)	**.021**	0.33 (0.10–0.56)	.237	0.40 (0.15–0.66)	.408

Values in parenthesis are 95%CI. ROC curves were calculated to assess the predictability of FVC% and DLco% decline from variations in QA patterns (Δ%Normal, Δ%GG, Δ%Reticular, Δ%GG+%Reticular). Progression was defined clinically as a FVC and DLco composite endpoint, consisting of either an FVC decline from baseline >10% or an FVC decline of 5–9% in association with a DLco decline of >15%. ROC curve of the composite functional endpoint is shown in Fig 3. Statistically significant p values (p<0.05) are in **bold**.

## Discussion

Our study provides evidence that QA can detect and objectively quantify SSc-ILD modification at CT during treatment and that QA has a weak-to-good concordance with semiQA performed by thoracic radiologists. Moreover, QA correlates better than semiQA with functional data, and both scores correlate with PA diameter at CT. Finally, our results show that changes in lung volumes measured by QA and not changes in QA patterns were able to predict changes in the composite functional respiratory endpoint with FVC% and DLco% and that clinical and quantitative radiological definitions of disease modification during treatment have a weak correlation.

QA revealed that GG and Reticular patterns characterized the SSc-ILD in all cases, with an apex-to-base crescendo of alterations for both patterns, in line with a previous study [4]. SemiQA had a good inter-reader concordance for evaluating the total disease extent and the Reticular pattern, and only fair for the GG pattern. The inter-reader concordance for total disease extent in our study was better than that reported by Moore et al. [24], likely because the reading of CT scans was performed by two dedicated chest radiologists in our study. Nonetheless, the inter-reader concordance for GG pattern was likely affected by the gradual contiguity or frank superimposition with Reticular pattern, as observed in most cases. This issue persists also with QA, as these regions represent mixed or transitional microscopic involvement [25]. However, QA defines each voxel as either GG or Reticular. This may lead semiQA to overestimate the superimposition between the two patterns and could in part explain not only the fair inter-reader concordance for GG pattern at semiQA, but also the weak concordance for Reticular pattern between semiQA and QA scores. This weak concordance was also observed considering the changes in Reticular pattern between baseline and follow-up. Indeed, we found no significant difference in %Reticular pattern at QA versus a slight difference in %Reticular pattern at semiQA as of global score and not evident at each lung level. Another possible cause of low concordance between semiQA and QA scores can be the different methodology, as QA is based on a 3D analysis of the entire lungs whereas semiQA is based on a 2D analysis performed at five predefined levels.

These methodological differences could in part justify the stronger correlation between PFT and QA in comparison to semiQA. In particular, in our study the strongest correlations were seen between %GG or %GG+Reticular and TLC% (r = -0.74 and -0.72, respectively) and between %GG or %GG+Reticular and FVC% (both r = -0.71). Our results showed overall better correlations between PFT and LTA than those reported in previous studies (r = -0.64 for FVC%) [10, 16]. The correlations between DLco% and the lung patterns of disease at both QA and semiQA were statistically significant, but low (r = -0.27 to -0.42) and slightly lower than that reported in a previous study on IPF patients analyzed by the same algorithm (r = -0.56) [16]. These correlations could mirror the combined vascular and inflammatory/fibrotic changes peculiar of scleroderma pathogenesis and may improve in next future with the segmentation of pulmonary vessels by dedicated software, especially if able to separate arteries from veins [26, 27].

The role of vascular impairment and endothelial dysfunction in SSc is fundamental in the pathogenesis of the disease [1]. The good correlations observed between SSc-ILD extent and PA diameter may reflect cor pulmonale from hypoxaemia (as seen in chronic obstructive pulmonary disease and obstructive sleep apnea), but may also support the hypothesis of vascular impairment associated to lung parenchymal changes of SSc-ILD. However, the pathophysiological mechanism underlying this relationship requires further investigation. A possible hypothesis could be the presence of a reduced vascular volume in fibrotic areas coupled by a thickening of the vessel intima in the spared areas, similarly to what has been speculated in IPF patients [28]. The involvement of pulmonary vessels elevates PA pressure and this is reflected on such a simple measurement as PA diameter. In a recent study the PA diameter revealed to be a good predictor of pulmonary hypertension (AUC of 0.88) in patients with SSc-ILD, even better than lung density measured as Perc85 [24]. A number of studies regarding the diagnostic potential of PA diameter as well as PA/Ao ratio have been extensively studied, using different cutoffs ranging from 25mm to 33mm. However, a recent meta-analysis on CT pulmonary artery measurements for the detection of pulmonary arterial hypertension showed that PA diameter measurement had 79% sensitivity and 83% specificity in diagnosing pulmonary hypertension, with a good overall accuracy, even better than echocardiography [23]. Furthermore, the meta-analysis revealed that PA diameter has better discriminatory ability than PA/Ao ratio in assessing pulmonary arterial hypertension [23], being in line with our results.

The strong correlation between PA diameter measurements and QA scores demonstrated in our study could help in distinguishing those patients with a more severe prognosis also when QA cannot be performed and it could further reveal the increase of PA pressure earlier than echocardiography [23].

PA diameter correlated also with traction bronchiectases, a radiological indirect sign of fibrosis. Traction bronchiectases are receiving growing attention in the medical community for their potential role in differentiating fibrotic and inflammatory ground-glass in ILD. Although the traction bronchiectases score is subjective, likewise any semiQA analysis, it has been demonstrated that determining the presence or absence of traction bronchiectases has a higher level of observer agreement than honeycombing [29]. The evaluation of traction bronchiectases is still demanded only to radiologst’ eye, as no accurate quantitative imaging tool is available nowadays to identify them and distinguish them from honeycombing, hyperlucent areas, or cysts.

This is the first study to evaluate QA performed by using Imbio LTA in patients with SSc-ILD during treatment follow-up. Changes in %Normal pattern were not accurate in predicting disease progression as defined by PFT. However, variation in lung volume calculated by LTA accurately predicted the composite functional respiratory endpoint with FVC% and DLco% (AUC of 0.74), which is the only accepted endpoint in SSc-ILD. It follows that functional data reflect lung volumes, but not disease extent and pattern type as radiologically quantified. While the clinical relevance of serial changes on CT is yet to be established, this clinical-radiological dissociation could improve the accuracy in objectively identifying CT serial changes in total disease extent as well as in different patterns, earlier than functional changes. In a previous study Moore et al. showed that a substantial deterioration in PFT (>30%) is needed to detect a major change at CT scoring performed by semiQA [30]. The advent of new expensive biological therapies for SSc created the need for an accurate evaluation of disease extent, pattern, and progression by using objective and reproducible tools. Imbio LTA performs a volumetric evaluation of parenchymal changes, showing modifications in lung patterns not revealed by PFT. The results of most important clinical trials on SSc-ILD are mainly based on PFT changes, demonstrating a limited although significant effect on FVC and DLco. Functional modifications during treatment are mandatory in evaluating these patients to assess patient prognosis [3]. However, the use of functional parameters as surrogate markers of disease progression has important limitations (i.e. significant intra-individual variability [31, 32], indirect estimation of fibrosis, the lack of evaluation of coexistent emphysema and/or pulmonary hypertension [33–35]). The position paper of the Fleischner Society on ILD recommended to investigate serial CT scans as part of a composite endpoint with trends in PFT and pointed at the multidisciplinary integration of CT with serial PFT as an area of future research [36]. Our study supports this perspective, with the suggestion of discussing QA results along with qualitative CT findings at the multidisciplinary meetings to assess more objectively the response to therapy and to ultimately personalize patient therapy. Indeed, QA provides consistent results that could be monitored and quantified, including not only an overall quantification of lung involvement, but also a precise definition of the lung patterns of disease [37]. Severity of traction bronchiectasis and increasing extent of honeycombing, unlike GG, have been reported as the strongest determinants of mortality in connective tissue diseases related ILD [29]. The recognition of the different patterns, including GG, as well as of the subtle changes by QA could be useful in targeting patient therapy and in assessing treatment efficacy, as recently suggested for IPF [37]. Diversification in treatment according to lung patterns could eventually result into a different prognosis, but nowadays this is just speculative.

The main limitation of this study is the relatively small cohort of subjects included to be considered representative of the whole SSc population. This aspect could have an impact on the power of our study. However, patients included were well characterized from both clinical and imaging perspectives with PFT, echocardiography, semiQA and QA. Furthermore, the pooling of CT data from one single center has the advantage to reduce the potential misinterpretation of QA derived from images obtained in different centers with different CT scanners and CT protocols. A second limitation regards semiQA, as it was performed by dedicated thoracic radiologists, who worked together at the same institution. This could have twisted the results towards a better inter-reader agreement, while in daily clinical practice general radiologists may have lower inter-reader concordance. A third limitation regards QA, which failed in a few cases (4/70 scans) despite observing all the technical parameters needed to perform QA. The fast technological advances may soon overcome this limitation, presenting already with a low rate.

### Conclusions

QA of lung parenchyma on chest CT scans can objectively quantify specific patterns of ILD changes during treatment in patients with SSc-ILD. Changes in QA patterns cannot predict disease progression as defined by functional changes, which only reflect lung volumes and not disease extent and pattern type. A single consistent measurement of PA diameter at CT reflects the interstitial involvement in patients with SSc, distinguishing patients with a more severe prognosis. Our results express the need of further investigation on this topic in both basic and clinical research. Basic research on pulmonary vessels in patients with SSc-ILD may deepen our knowledge on the endothelial dysfunction in SSc, whereas clinical research could benefit from the use of objective imaging biomarkers as endpoints to eventually treat patients with a more personalized approach.
